# Deubiquitinating Enzymes and Bone Remodeling

**DOI:** 10.1155/2018/3712083

**Published:** 2018-07-08

**Authors:** Yu-chen Guo, Shi-wen Zhang, Quan Yuan

**Affiliations:** State Key Laboratory of Oral Diseases, National Clinical Research Center for Oral Diseases, West China Hospital of Stomatology, Sichuan University, Chengdu 610041, China

## Abstract

Bone remodeling, which is essential for bone homeostasis, is controlled by multiple factors and mechanisms. In the past few years, studies have emphasized the role of the ubiquitin-dependent proteolysis system in regulating bone remodeling. Deubiquitinases, which are grouped into five families, remove ubiquitin from target proteins and are involved in several cell functions. Importantly, a number of deubiquitinases mediate bone remodeling through regulating differentiation and/or function of osteoblast and osteoclasts. In this review, we review the functions and mechanisms of deubiquitinases in mediating bone remodeling.

## 1. Introduction

The human skeleton undergoes continuous bone remodeling throughout a lifetime [1]. This process initiates with the destruction of mineralized bone, followed by the formation and mineralization of a new bone matrix [1, 2]. This critical process adapts bone architecture and strength to mechanical needs as well as growth. Meanwhile, it repairs microdamage of bone structure and maintains calcium homeostasis [1, 2]. Thus, bone remodeling is pretty important to general health.

To maintain bone homeostasis, bone remodeling is carried out by three main cell lineages: osteoclasts, multinucleated cells differentiate from macrophages and monocytes in the human hematopoietic lineage, resorb mineralized bone, and initiate the bone remodeling cycle [3]; osteoblasts, differentiate from mesenchymal stem cells (MSCs), deposit, and mineralize a new bone matrix [4]; osteocytes, which are the most common cells divided from osteoblasts, serve as a sensing and information transfer system [2]. These cells constitute the basic multicellular unit (BMU) that carries out the bone remodeling cycle. Based on current knowledge, bone remodeling mainly involves the following phases: formation of osteoclasts and resorption of bone, which initiates the cycle; completion of bone resorption followed by recruitment and differentiation of MSCs into osteoblasts; and bone formation mediated by osteoblasts [2]. Thus, the differentiation, function, and interaction of these BMU cells are critical to regulate bone remodeling and maintain bone homeostasis.

Osteoclasts that trigger the bone remodeling cycle are formed by the fusion of mononuclear progenitors in osteoclastogenesis [2]. They exist in a motile state during which they migrate from the bone marrow to the resorption site or a resorptive state performing their bone resorption function [5]. Osteoclasts are derived from the hematopoietic lineage and regulated by several factors [6]. Among these factors, M-SCF and RANKL produced by marrow stromal cells and osteoblasts are essential to promote osteoclastogenesis [2]. Osteoblasts play a key role in bone formation. They arise from MSCs and their differentiation is mainly regulated by transcription factor RUNX2 at the early time. They begin to express osteoblast phenotypic genes and synthesize the bone matrix at a later stage [7, 8]. Then osteoblasts are embedded into the bone matrix as osteocytes or die at the end of their destiny [9]. Several mechanisms including transcription factors, growth factors, hormones, and the extracellular matrix regulate these stages [7, 10]. In the last few years, significant findings have unveiled the mysterious role of the ubiquitin-dependent proteolysis system (UPS) in regulating differentiation and function of osteoclasts as well as osteoblasts [11–13].

## 2. Ubiquitin-Dependent Proteolysis System

Ubiquitin is a highly conserved protein which is made up of 76 amino acids. It is linked to the lysine side chains of target proteins, which results in monoubiquitination or polyubiquitination of the protein. Polyubiquitylated proteins are degraded within a cylindrical multiprotein complex that is named proteasome [14, 15], while monoubiquitination has a variety of ends except proteasomal degradation [14, 15]. For example, the adapter protein TRAF6 contains the RING finger domain which could generate nondegradative K63-linked ubiquitin and contribute to form signaling complexes [16]. This is important to mediate RANK/TRAF6 signaling [17]. To successfully add ubiquitin to target protein, three enzymes involved in this process are essential. The E1 enzyme that recruits ubiquitin is named ubiquitin-activating enzyme. The E2 enzyme, called ubiquitin-conjugating enzyme, transfers the ubiquitin to protein. The E3 enzyme, also known as ubiquitin ligase, acts as a scaffold protein which interacts with the ubiquitin-conjugating enzyme and transfers ubiquitin to protein [18]. Consequently, the UPS affects multiple processes such as protein degradation, cell death, vesicular trafficking, signal transduction, DNA repair, and stress responses [11, 14, 15, 19–23].

The ubiquitin-dependent proteolysis system plays an important role in mediating bone remodeling. Initially, by inhibiting the proteasomal function through proteasome inhibitor I (PSI), study demonstrated that the UPS is an important regulator of bone turnover and chondrogenesis [24]. And administration of proteasome inhibitor Bortezomib induced MSCs to undergo osteoblastic differentiation partially by modulation of RUNX2 in mice [25]. As a clinically available proteasome inhibitor used in myeloma, Bortezomib is also reported to promote osteoblastogenesis as well as inhibit bone resorption in clinical studies [26, 27]. Following studies demonstrated that these effects are mainly mediated by inhibiting the proteasomal degradation of important proteins, which regulate osteoblast function such as *β*-catenin [28] and Dkk1 [26]. Another protein stabilized by proteasome inhibitor is Gli2, which promotes bone formation through upregulating bone morphogenetic protein-2 (BMP2) [29, 30].

To date, studies investigating ubiquitin ligase and bone remodeling have demonstrated that several E3 ubiquitin ligases take part in regulation of bone metabolism. For example, the first known ubiquitin ligase affecting bone formation is Smuf1. Smurf1 has been proved to mediate RUNX2 degradation, resulting in downregulated osteoblast differentiation and bone formation [31–35]. Smurf1 also regulates the degradation of Smad1 and downregulates BMP-induced osteogenic differentiation of MSCs [35–37]. Moreover, Smuf1 mediates JunB, MEKK2, and other molecule proteasomal degradation, which causes the inhibition of osteoblast differentiation [32, 38, 39]. Another important ubiquitin ligase which regulates osteoblastogenesis is Cbl. It controls osteoblastogenesis by controlling the ubiquitination and degradation of receptor tyrosine kinases (RTKs), including IGFR, FGFR, and PDGFR [40–43]. Cbl also interacts with Pl3K to regulate bone formation [44–47]. Besides, Itch and Wwp1 are demonstrated to regulate osteogenesis by promoting RUNX2 degradation [48, 49]. On the other hand, E3 ligases also influence osteoclastogenesis and bone resorption. The E3 ligase LNX2 promotes osteoclastogenesis through M-SCF/RANKL signaling as well as the Notch pathway [13]. Another ubiquitin E3 ligase RNF146 inhibits osteoclastogenesis and cytokine production via RANK signaling [50]. As there are over 600 E3 ligases expressed in the human genome, lots of E3 ligases are found to regulate bone remodeling by governing BMU cell differentiation and function.

## 3. Deubiquitinases

Like other posttranslational modifications, the process of ubiquitination is reversible by the function of deubiquitinases (DUBs) which remove monoubiquitin or polyubiquitin chains from such ubiquitin-modified proteins [51]. Ubiquitin itself is a long-lived protein [52, 53]; thus, it is necessary to remove ubiquitin from proteins for maintaining a sufficient pool of free ubiquitin in the cell to sustain a normal rate of proteolysis. As key hydrolytic emzymes, DUBs hydrolyze the peptide bond that links target protein and ubiquitin [54]. Deubiquitinases are modular proteins which contain catalytic domains, ubiquitin binding domains, and protein-protein interaction domains. Such modules make positive contribution to the recognition of and binding to various chain linkages [55]. To date, about 100 DUBs have been reported to be encoded by the human genome [56, 57] (Table 1). According to their catalytic domains, these DUBs can be classified into five families including 4 thiol protease DUBS (USP, UCH, OUT, and Josephin) and 1 ubiquitin specific metalloproteases (JAMM) [54].

Deubiquitination has also been reported to be involved in many cellular functions, including DNA repair, protein degradation, cell cycle regulation, stem cell differentiation, and cell signaling [58–69]. Besides, a number of articles demonstrated that DUBs are essential for bone remodeling through regulating related BMU cell differentiation and function [69–78].

### 3.1. Ubiquitin-Specific Protease (USP) and the Bone

The ubiquitin-specific protease family, which contains 56 members in human, is the largest and most diverse family of the DUB families. Consisting of 6 conserved motifs, these USP catalytic domains vary between 295 and 850 residues [57]. Within these 6 motifs, there are two well-conserved motifs that are named Cys-box and His-box. They contain all the necessary catalytic residues [55, 57]. The structure of USP7 is the first well described with three subdomains resembling like a right hand [79]. The thumb and the palm contain Cys-box and His-box, respectively. The cleft between them is the catalytic center. The finger domains can interact with ubiquitin to transfer its C-terminal to the cleft [79]. Then USP5 showed us how UBL domains inserted into a single USP domain to provide additional ubiquitin binding sites which make it possible for the enzyme to bind and disassemble poly-Ub chains [80].

USP is reported to be involved in many cell functions. Most importantly, as the largest family of DUB, USPs are found to regulate bone remodeling by controlling the function of osteoblast, osteoclast, and even PTH.

#### 3.1.1. USP and Osteoblast

USP4 is found to regulate osteoblast differentiation through the Wnt/*β*-catenin signaling pathway [70]. The canonical Wnt signaling pathway is essential for osteoblast differentiation and bone formation. A study demonstrates that USP4 inhibits this pathway by deubiquitinating the polyubiquitin chain from Dvl, resulting in inhibiting of Wnt signal and decreased osteoblast differentiation and mineralization [70]. USP4 also deubiquitinates other Wnt signaling components such as Nik and TCF4 [81]. There are also findings indicating that USP4 positively controls *β*-catenin stability by deubiquitinating, leading to the activation of Wnt signaling [82, 83]. Thus, further researches focusing on USP4 and the Wnt signaling pathway are strongly needed. Besides, USP4 is an important TGF/BMP signaling pathway regulator [69]. After phosphorylation by AKT, USP4 associates with and deubiquitinates ALK5, leading to upregulation of TGF*β* signal [84]. In accordance with this finding, USP4 is also reported to interact with Smurf2 and Smad7 [85]. Furthermore, USP4 stabilizes Smad4 through inhibiting its monoubiquitination and enhances activin as well as BMP signaling [86]. Because TGF/BMP signaling plays a pivotal role in osteogenic differentiation of MSCs and bone formation [87], future studies may reveal the essential role of USP4 in control osteoblast differentiation and function through regulating this signaling.

Recently, a study has revealed that USP7 is related to osteogenic differentiation of human adipose-derived stem cells (hASCs) [71]. Like MSCs, hASC is also a stem cell with multilineage differentiation ability, including osteogenic differentiation. USP7 depletion leads to impaired osteogenic differentiation of hASCs. Overexpression of USP7 upregulates hASC osteogenesis. Moreover, knockdown of USP7 results in impaired bone formation *in vivo* [71]. USP7 acts to ubiquitinate and stabilize PHF8, an epigenetic factor which is essential for stem cell fate determination [88, 89]. Importantly, PHF8 triggers osteogenic differentiation of BMSCs [90]. Thus, the possible mechanism by which USP7 upregulates osteogenic differentiation of hASCs might be that USP7 stabilizes PHF8. A further study is still needed to uncover the actual mechanisms.

USP15, which is highly similar with USP4 [69], also is involved in Wnt signaling and bone formation [91]. USP15 stabilizes *β*-catenin and enhances Wnt signaling. These processes are initiated by FGF2, which activates MEKK2, causing recruitment of USP15 [91]. USP15 is involved in the TGF/BMP signaling pathway through connecting with ALK3, ALK5, and monoubiquitylated R-SMADs [92–94]. Future studies might reveal the relationship among USP15, TGF/BMP signaling, and osteoblast function.

Interestingly, USP9x, also known as fat facets in mouse (FAM), is closely associated with the TGF/BMP cell signaling pathway, a key signal pathway related to osteogenesis and bone formation. USP9x hydrolyzes Smad4 monoubiquitination [95–97], enhancing TGF-*β* signal. Moreover, USP9x interacts with the WW domain of Smurf1 and stabilizes it [72]. As told above, Smurf1 plays a pivotal role in osteogenic differentiation and bone formation [31–37]. Likely, USP11 is also involved in the TGF/BMP signaling pathway by deubiquitylating ALK5 [98]. These data suggest the potential direction of future studies.

#### 3.1.2. USP and Osteoclast

USPs not only control osteogenic differentiation and bone formation but also regulate osteoclast differentiation and function. For example, CYLD inhibits osteoclastogenesis via downregulating RANK signaling [99]. CYLD deubiquitylates TRAF6, which transduces the RANK-mediated signal [99]. By this mechanism, CYLD inhibits osteoclast differentiation, leading to severe osteoporosis *in vivo* [99]. Using proteasome inhibitors, another study also emphasizes the key role of CYLD in osteoclast formation and function [100]. Furthermore, SCF*β*-TRCP controls the degradation of CYLD itself, which pinpoints SCF*β*-TRCP/CYLD as a pivotal modulator of osteoclastogenesis [101].

USP18 inhibits osteoclastogenesis in mice [77]. IFN signaling negatively influences osteoclastogenesis [102]. Type I IFN stimulates ISG, a ubiquitin-like protein, to express and conjugate to its target ISGylation [103]. Research data demonstrates that USP18 is a negative regulator of IFN signaling via deconjugating ISGylation [104–106]. USP18 deficiency leads to increased RANKL-mediated osteoclastogenesis, resulting in osteopenia phenotype *in vivo* and *in vitro* [77].

USP15, which regulates osteoblast function and bone formation, is connected to osteoclast function too [76]. USP15 is the key DUB which cooperates with CHMP5 to stabilize I*κ*B*α*, leading to decreased RANKL-mediated NF-*κ*B activation and osteoclast differentiation [76]. Taken together, USP15 might be an essential regulator of bone remodeling.

#### 3.1.3. USPs and PTH

In addition to some USPs that regulate osteoblast and/or osteoclast function, there are also some other USPs which collaborate with PTH to influence bone turnover. USP2 was found to be stimulated by PTH in the bone. These osteotropic agents, including PTH, PTHrP, and PGE2, can stimulate USP2 expression selectively in the bone through the PKA/cAMP pathway [107]. A further study revealed that PTH (1-34) could upregulate the expression of USP2 and promote PTHR deubiquitination as well as stabilization [108]. Recently, research data have demonstrated that USP2 is necessary for PTH (1-34) to induce osteoblast proliferation [109]. These findings emphasize the importance of USP2 in PTH mediating anabolic action of bone formation. Another study focusing on the relationship between miRNAs and the PTH level in end-stage renal disease patients demonstrates the close connection between miR-3680-5p and the PTH level. Interestingly, the target genes of miR-3680-5p are *USP2*, *USP6*, *USP46*, and *DLT*, all of which are members of the UPS [110]. Taken together, USPs may regulate bone turnover via the influence of PTH-associated bone formation. In the future, studies about the details of this interesting mechanism will be the focus.

### 3.2. Ubiquitin C-Terminal Hydrolase (UCH) and Bone Formation

The members of the UCH family are several thiol proteases which contain a 230-residue domain as a catalytic core, an N-terminal, and followed by C-terminal extensions which mediate protein to protein interactions sometimes [54]. In human, four UCHs are grouped into smaller UCHs (UCH-L1 and UCH-L3) that prefer to cleave small leaving groups from the C-terminal of Ub and larger UCHs (UCH37 and BAP1) that hydrolyze polyubiquitin chains [54].

Like USPs, UCHs are also reported to have multiple functions [111–113]. Importantly, UCH-L3 deubiquitylates Smad1 and enhances osteoblast differentiation [73]. UCH-L3 physically interacts with Smad1 and stabilizes it by deubiquitylating its polyubiquitin. UCH-L3 promotes the differentiation of osteoblast from C2C12 cells, while knockdown of Uch-l3 delays osteoblast differentiation [73]. Likely, UCH37 is found to connect to Smad7 and reverse Smurf-mediated ubiquitination [114]. Moreover, UCH37 affects TGF-*β* signaling by connecting to ALK5 [115]. In all, UCH37 influences TGF-*β* signaling that suggests the role of UCH37 in regulating osteoblast differentiation and function.

### 3.3. Ovarian Tumor (OTU) and the Bone

The OUT family was identified based on their homology to the ovarian tumor gene [54]. In human, there are 15 OUTs that are usually grouped into three subclasses: the otubains or OTUBs, the OTUs, and the A20-like OTUs [54].

Among numerous functions of OTUs [116–120], A20 demonstrates the ability to regulate osteoclastogenesis [78, 121, 122]. Bacterial lipopolysaccharides and RANKL induce human peripheral blood mononuclear cells to express A20, which is associated with TRAF6 and NF-*κ*B degradation. Knockdown of A20 results in increased bone resorption [121]. A20 has anti-inflammatory effects as well as antiosteoclastogenic effects [78, 122], which is mainly governed by its attenuation of NF-*κ*B signaling through regulating IKKs [123]. Moreover, A20, which is recruited by Smad6 to TRAF6, plays an important role in inhibition of noncanonical TGF-*β* signaling [124], indicating its possible regulation of osteoblastogenesis via this main pathway. Besides, like A20, OTUB1 is also involved in TGF-*β* signaling through deubiquitination of the p-SMAD2/3 complex [125]. Studies focusing on the function of OTUs in osteoclast differentiation and function will reveal more details about the second largest DUB family.

### 3.4. JAB1/MPN+/MOV34 (JAMM) and the Bone

There are eight JAMM domain proteins in human, including PRPF8 without protease activity [51, 54]. All of JAMM DUBs are found with subunit complexes of proteasome, such as the proteasome 19S lid complex (POH1/hRpn11) and the COP9 signalosome (CSN5/Jab1) [54]. As an endopeptidase, RPN11 functions to cleave polyubiquitin chains from substrates [126] While CSN5/Jab1 hydrolyzes the ubiquitin-like modifier Nedd8 [127], POH1 enhances osteoclast differentiation and RANKL signaling via regulating Mitf, an important regulator of osteoclast differentiation which required gene expression [128]. MYSM1, a member of the JAMM family, is a histone DUB which specifically deubiquitinates histone 2A [129]. MYSM1 deficiency leads to decreased bone mass. MYSM1 deficiency results in impaired osteogenic differentiation of both mouse MSCs and MC3T3-E1 cell [75]. Recently, study demonstrates that MYSM1 deficiency impairs the potential for primary osteoblasts to differentiate into mature osteoblasts. Meanwhile, MYSM1 knockout reduces the proliferation of osteoclast progenitor and the osteoclast resorption activity [130]. With further studies that might uncover the detailed mechanisms of MYSM1 regulating osteoblast and osteoclast differentiation, this DUB may be a potential therapeutic target for related bone diseases.

The last member of DUBs is Josephin. There are four proteins belonging to this family, including Ataxin-3, Ataxin-3L, Josephin-1, and Josephin-2 [54]. Unfortunately, current studies have not reported the relationship between Josephin DUBs and skeleton cell differentiation and function. Further studies about the members of Josephin may find novel mechanisms by which these DUBs regulate osteoblast and osteoclast functions.

## 4. Perspective

The ubiquitin-dependent proteolysis system is crucial to cellular functions including skeleton cell functions. The roles of ubiquitin ligases in regulating osteoblast and osteoclast differentiation are well studied, while studies about deubiquitinating enzymes and skeleton cell differentiation are still lacking. In order to delineate the ubiquitin-dependent proteolysis system to regulate bone remodeling, it is important to establish our knowledge about DUBs and bone remodeling. To date, several DUBs are found to regulate osteoblast function (USP4, USP7, USP9x, USP15, UCH-l3, and MYSM1) and osteoclast function (CYLD, USP15, USP18, A20, and POH1) (Table 2). But the mechanisms by which these DUBs regulate skeleton cell functions are not exhaustively described. Future studies should find more DUBs that are involved in BMU cell function and bone remodeling. Importantly, the major challenge is to well describe the actual mechanisms behind these phenotypes. With these novel findings, drugs targeting these DUBs will be designed to treat related skeleton diseases.

## Figures and Tables

**Table 1:** Members of deubiquitinases.

Family	Members
USP	USPL1, CYLD, USP1, USP2, USP3, USP4, USP5, USP6, USP7, YSP8, USP9x, USP10, USP11, USP12, USP13, USP14, USP15, USP16, USP17L2, USP18, USP19, USP20, USP21, USP22, USP23, USP24, USP25, USP26, USP27, USP28, USP29, USP30, USP31, USP32, USP33, USP34, USP35, USP36, USP37, USP38, USP39, USP40, USP41, USP42, USP43, USP44, USP45, USP46, USP47, USP48, USP49, USP50, USP51, USP52, USP53, USP54
OTU	OTUB1, OTUB2, OTUD1, OTUD3, OTUD4, OTUD5, OTUD6A, OTUD6B, OTU1, HIN1L, A20, Cezanne, Cezanne2, TRABID, VCPIP1
UCH	UCH-L1, UCH-L3, UCH37/UCH-L5, BAP1
Josephin	ATXN3, ATXN3L, JOSD1, JOSD2
JAMM/MPN+	BRCC36, CSNS, POH1, AMSH, AMSH-LP, MPND, MYSM1, PRPF8

**Table 2:** Deubiquitinases and bone remodeling.

Family	Name	Function	Mechanism	Ref.
USP	USP4	Inhibits osteoblast differentiation and mineralization	Regulates Wnt signaling by deubiquitinating Dvl, Nik, TGF4, and *β*-catenin; may regulate TGF/BMP signal	[67–79, 81–85]
	USP7	Enhances osteogenic differentiation of hASCs	Stabilizes PHF8 that triggers osteogenic differentiation of BMMSCs	[69, 86–88]
	USP15	Enhances osteoblast-mediated bone formation	Regulates Wnt signaling via deubiquitinating *β*-catenin	[67, 89–92]
	CYLD	Inhibits osteoclastogenesis	Regulates RANK signaling through deubiquitinating TRAF6	[97–99]
	USP18	Inhibits osteoclastogenesis	Regulates IFN signaling by deconjugating ISGlation	[75, 102–104]
	USP15	Inhibits osteoclastogenesis	Stabilizes I*κ*B*α*, leading to decreased RANKL-mediated NF-*κ*B activation	[74]
	USP2	Necessary for PTH (1-34) to induce osteoblast proliferation	Upregulated by the PKA/cAMP pathway and stabilizes PTHR	[105–108]
UCH	UCH-L3	Increases osteoblast differentiation	Interacts with Smad1 and stabilizes it by deubiquitylating its polyubiquitin	[71]
OTU	A20	Inhibits osteoclastogenesis	Regulates RANK signaling by controlling TRAF6 and NF-*κ*B degradation	[76, 119–123]
JAMM	POH1	Enhances osteoclast differentiation	Regulates Mitf	[126]
	MYSM1	Enhances osteogenic differentiation	—	[73, 127, 128]
